# The changing landscape of clinical trials in Australia

**DOI:** 10.5694/mja2.52059

**Published:** 2023-08-13

**Authors:** Anna Lene Seidler, Melina L Willson, Mason Aberoumand, Jonathan G Williams, Kylie E Hunter, Angie Barba, R John Simes, Angela Webster

**Affiliations:** ^1^ NHMRC Clinical Trials Centre University of Sydney Sydney NSW

**Keywords:** Clinical trials as topic, Research design, Registries

Examining the clinical trials landscape in Australia is important for governance and to expand knowledge about trial activity to health professionals, the public and funders. There is a strong history of Australian trials addressing important health care questions by covering a range of diseases, patient groups, prevention and treatment modalities.^1^ Trial results can drive change by informing best practice in health care and future research.

In this perspective, we present key findings from a comprehensive report of Australian clinical trial activity from 2006 to 2020.^2^ We describe characteristics and trends of Australian trials, make international comparisons, and outline areas for further enquiry or support. Data are sourced from the Australian New Zealand Clinical Trials Registry (ANZCTR) that captures over 95% of registered trials occurring in Australia through trial registration and data feed from ClinicalTrials.gov.^2^ The methods are detailed in the Supporting Information.

## Australian trial activity compares favourably internationally

Australia has a highly active trials community, exemplified by the number, breadth and sponsorship of trials (Box 1). From 2006 to 2020, over 18 000 trials recruiting participants in Australia were registered, 40% of those in the period 2016–2020. This includes Australia‐only (12 775 trials, 69%) and multinational trials (5678 trials, 31%). These trials planned to recruit a total of 8.7 million people from 2006 to 2020, albeit this includes participants in Australia and internationally.

Box 1 **Overall characteristics of Australian clinical trials, 2006–2020**

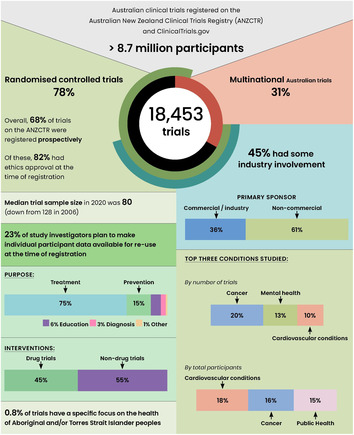

Australian trial activity (number of trials per capita) compares favourably with other OECD countries, with more activity in Australia than in France, Germany, and the United States (Box 2). Few countries have more activity than Australia, including Belgium and Denmark. An even further improvement of Australia's trial activity could be achieved by better integration of trial research in routine health care. The implementation of the Australian Commission on Safety and Quality in Health Care National Clinical Trials Governance Framework^5^ in public and private health services is expected to address this need. Streamlining of approval processes for trial start‐up is anticipated through the proposed National One Stop Shop platform.^6^

Box 2 Registered study activity per capita for Australia and selected countries, 2006–2019*

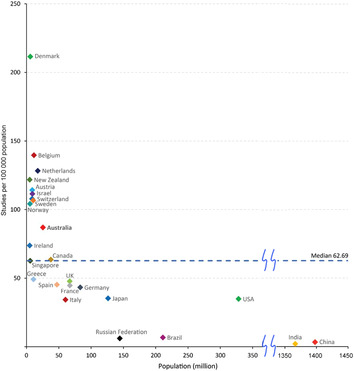

* Study activity data derive from the WHO Global Observatory on Health Research and Development;^3^ population data derive from the World Bank.^4^

The impact of the COVID‐19 pandemic on trial activity is complex, with further in‐depth analysis desirable. Data from the first 11 pandemic months suggest a decline in the number of Australian trials starting in 2020 (17% fewer than in 2019). Initial reports indicate a similar decline of non‐COVID‐19 trials in Europe and the US in 2020,^7^, ^8^ and a nominal reduction in new trials internationally in 2021.^3^

## Smaller trials over time

Although there has been an increase in the number of Australian trials over time, the number of people anticipated to enrol, or actually enrolled, in these trials has gradually declined from a median sample size of 128 participants in 2006 to 80 in 2020 (Box 3). Internationally, similar trends have been observed (eg, in the US).^9^ Several factors may explain these smaller sample sizes. First, there has been an increase in registrations of earlier phase drug trials, which has grown substantially from 9% in 2006 (35/390) to 40% in 2020 (222/557), while the proportion of phase 3 and 4 trials has halved from 50% in 2006 (196/390) to 26% in 2020 (143/557; Box 4). This might reflect changing registration practices resulting in more complete capture of early phase trials in recent years. Second, anticipated operational challenges such as delays in starting the trial, difficulty in recruiting participants, and insufficient funding can contribute to small sample sizes. Other factors could include greater targeting of restricted populations (eg, recruitment by biomarker status), or greater efficiencies in design, such as increasing reliance on surrogate outcomes (a potentially less relevant outcome requiring smaller sample sizes), which might lead to fewer trials informing patient care.^10^ Overall, it is key to prioritise quality over quantity by improving trial methodology and doing trials strategically to maximise health impact.^11^, ^12^ Interrogating this could be the focus of future work.

Box 3 Changes in sample size (median and interquartile range), 2006–2020*

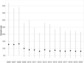

* Sample size refers to the actual sample size (if reported); otherwise, anticipated sample size.

Box 4 **Proportions of phases of drug trials, 2006–2020**

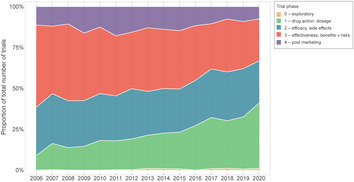

## Broad scope of health conditions and interventions addressed by variety of funders

From 2006 to 2020, the most frequently studied health conditions based on number of trials were cancer (20%, 3666/18453), mental health (13%, 2413/18453) and cardiovascular diseases (10%, 1841/18453; Box 5). In the last five years, the number of trials focused on neurological conditions and public health topics have overtaken trials on cardiovascular diseases, though the difference is small. We assessed how trial activity, defined by the total number of trial participants per condition, relates to national burden of disease (measured by disability‐adjusted life‐years).^13^ Compared with burden of disease, cancer and cardiovascular trials seem to be well represented, whereas mental health, neurological and musculoskeletal trials seem to include fewer participants than expected.

Box 5 **Health conditions studied in Australian clinical trials, 2006–2020**

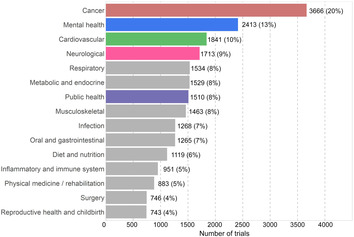

Most Australian trials examine treatment strategies (75%, 13716/18375), followed by prevention (15%, 2678/18375). Although drugs are the most studied intervention type, with 45% of trials (8292/18453) from 2006 to 2020, their proportion has declined (55% in 2006; 33% in 2020). The 77% of non‐drug trials registered in 2020 included prevention, devices, behaviour, lifestyle, and other non‐drug treatments.

The economic investment in Australian trials is substantial, with an estimated $1.4 billion of public and private funding committed in 2019.^14^ Trials in Australia are conducted through universities, hospitals, individuals, government, charities, and commercial entities. Just under half (45%) of trials registered between 2006 and 2020 declared industry involvement (ie, industry funding, sponsorship/collaboration, or combination). Industry involvement typically also means industry funding; 93% of trials with industry involvement were also funded by industry.^15^ Trials with industry involvement differ in their characteristics from non‐industry trials; for instance, they are more likely to study treatments rather than prevention or education interventions.^15^ Targeted public funding schemes (eg, Medical Research Futures Fund Australia) can support the prioritisation of diverse research themes such as rare diseases and public health interventions. Thus, a combination of public‐ and industry‐funded trials is important to sustain the breadth of trials in Australia.

## Understanding diversity and inclusion in trials hampered by poor availability of metrics

Trials in diverse settings and involving diverse participants are most likely to produce generalisable results to inform care.^16^, ^17^ Yet, we are unable to provide an overview of trial participation in Australian trials based on sex, gender, cultural, ethnic and linguistic diversity because this information is not collected consistently in most public resources, including trial registries. Two actions are in development: first, the ANZCTR is scoping options to capture data on sex and gender diversity in a standardised way; second, recommendations for consistent methods of collecting information about cultural, ethnic and linguistic diversity in trials are outlined by the Australian Clinical Trials Alliance.^17^ These issues also apply to other underrepresented groups (eg, older adults, those with co‐morbid illness or rare diseases). Representation and transparent reporting of outcomes for diverse groups in trials are crucial to ensure health care meets the needs of all population groups. An important area to consider when addressing representativeness in Australian trials is involving Aboriginal and Torres Strait Islander peoples. We were unable to assess overall participation in trials in Australia since this information was not explicitly recorded on the ANZCTR or other trial registries. However, we can estimate the number of trials focusing on the health of Aboriginal and Torres Strait Islander peoples exclusively by searching trial eligibility criteria. We found that these trials have slightly increased over time; from two trials (0.3% of all trials) in 2006 to 16 trials (1% of all trials) in 2019. Trials focusing exclusively on health of Aboriginal and Torres Strait Islander peoples were more likely to investigate prevention over treatment than other Australian trials (prevention: 36% *v* 15%; treatment: 52% *v* 75%)^18^ and were less likely to be funded by industry (7% *v* 17%).^17^ Engagement with Aboriginal and Torres Strait Islander communities would be a first step to understand how information on trial participation might be documented and analysed in line with the principles of Indigenous data sovereignty.^19^

## Hesitancy in uptake of opportunities for open science

The open science movement aims to make research processes, data, and dissemination accessible and transparent. This increases the efficiency and effectiveness of scientific research in improving human health outcomes and encourages public trust in science. In this spirit and in line with international standards, trial registries have the capacity for trialists to report results and trial protocols,^20^ the latter being critical for interpreting results. In contrast to other registries operating in similar regulatory frameworks, such as the ISRCTN registry in the UK,^21^ uptake was low for the ANZCTR (ie, reporting results: ANZCTR, < 13% of registered trials *v* ISRCTN, 54%; reporting protocols: ANZCTR, < 8% *v* ISRCTN, 22%). Prospective trial registration is another important cornerstone of the open science movement which the ANZCTR is promoting and facilitating, with in‐depth analyses published elsewhere.^22^, ^23^

Another aspect of the open science movement is data sharing. This has many advantages — new research questions can be answered without new recruitment, and more nuanced data can be accessed to inform clinical practice guidelines. Since October 2018, the International Committee of Medical Journal Editors and the World Health Organization (WHO) have required trialists to state whether they plan to share de‐identified data at the time of registration.^20^, ^24^ Since then, only 485 (23%) of 2143 Australian trials have indicated the intention to share data. Internationally, rates are similar, with 23% of trials on the WHO trials platform intending to share data.^25^ Detailed analyses of barriers and pathways for data sharing are published elsewhere.^26^ Building the right infrastructure and regulatory frameworks to facilitate data on‐use is important. The Health Studies National Data Asset program^27^ coordinated by the Australian Research Data Commons has delivered such infrastructure by building a network of Australian researchers with the skills and processes to share data. This model can be adapted by other countries to develop initiatives for better international data sharing and collaboration. Putting in place measures to increase the uptake of open science, including protocol publication, results reporting and data sharing, is critical to maximise knowledge gain from trials and ensure health care decisions are based on a complete and high quality evidence base.

## Conclusion

This snapshot on the trials landscape in Australia shows a healthy trial activity compared with other countries. There is diversity in health topics and trial phases being investigated, enabled by a range of funding sources. We have highlighted areas requiring further investigation, including the impact of the COVID‐19 pandemic on trials, and trials becoming smaller over time. Areas in need of improvement include reporting on the inclusion of priority groups, and increasing results reporting and data sharing via trial registries. Trial registries are a unique resource that can inform future research prioritisation, facilitate open science, and help increase awareness of trials for Australian health care workers, researchers, and the public. The full report is available online.^2^

## Open access

Open access publishing facilitated by The University of Sydney, as part of the Wiley ‐ The University of Sydney agreement via the Council of Australian University Librarians.

## Competing interests

No relevant disclosures.

## Provenance

Not commissioned; externally peer reviewed.

## Supporting information


Supporting Information
