# Supplementary material for: The changing landscape of clinical trials in Australia
Source: Med J Aust. 2023 Aug 13;219(5):192–6. doi: 10.5694/mja2.52059 (PMC10952960; doi:10.5694/mja2.52059)
Supplement: Supplementary file 1 — Supporting Information [file MJA2-219-192-s001.pdf]

The Medical Journal of Australia

Supporting Information

Supplementary methods

This appendix was part of the submitted manuscript and has been peer reviewed. It is posted as supplied by the authors.

Appendix to: Seidler AL, Willson ML, Aberoumand M, et al. The changing landscape of clinical trials in Australia. *Med J Aust* 2023; doi: 10.5694/mja2.52059.

Definitions

'Australian' clinical trials: studies with Australia listed as a recruitment country. These trials may be recruiting within Australia at a single site, multiple sites, or be part of a multinational study with multiple recruitment countries in addition to Australia.

Clinical trials: research studies that recruit people to test new 'interventions'. These can be drugs, devices, vaccines, surgery, behavioural therapies, preventive care changes, other interventions or combinations of interventions, given to individuals or applied to systems, that are designed to help improve human health. The World Health Organization (WHO) defines a clinical trial as 'any research study that prospectively assigns human participants or groups of humans to one or more health-related interventions to evaluate the effects on health outcomes'.

Country-specific clinical trial activity: estimated number of studies per 100,000 people based on the population size for each country divided by the number of registered studies (interventional and observations).

Industry involvement: industry is listed as the funding source, primary sponsor, secondary sponsor, other collaborator, or a combination of any of the aforementioned. Industry involvement usually refers to some level of industry funding.

Multinational trials: trials with recruitment sites in Australia and overseas.

Number of participants: includes the planned recruitment or, if available, actual number of participants of such trials both in Australia and overseas.

Phase of trial: the research steps used to investigate new interventions, most commonly for drugs, with each phase designed to address a specific question.

Sample size: the target or actual sample size (depending on the recruitment status at the time of registration and subsequent updates).

Year: a trial's year of registration, i.e. the year the study was approved for listing on the ANZCTR or ClinicalTrials.gov. This does not necessarily reflect the year the trial started.

Data source

Data are sourced from the ANZCTR and direct data feeds of Australian recruitment sites from the US-based registry ClinicalTrials.gov. This means that over 95% of registered trial activity in Australia is captured. The other 4% of Australian trials are registered on one of the other 17 WHO primary registries.

Trials may be registered on more than one register, although the ANZCTR's process aims to avoid this where possible: 253 trials (less than 1.5% of the total) are known to be registered on both the ANZCTR and ClinicalTrials.gov, and therefore may be counted twice in some figures.

Methods

Interventional studies were identified using the 'Study type' field on both registries. Those that selected 'Interventional' for this field were extracted from ANZCTR, and those that selected either 'Interventional' or 'Expanded Access' were extracted from ClinicalTrials.gov.

Studies that did not involve an intervention, but were purely observational in nature, were excluded from analyses (unless otherwise stated in the Report).[1]

All available data fields for interventional studies were extracted from both registries. The data fields collected by ClinicalTrials.gov are slightly different from those collected by ANZCTR (see https://prsinfo.clinicaltrials.gov/definitions.html). Where possible, ClinicalTrials.gov fields were mapped to match ANZCTR fields, to enable synthesis of data. Details of data mapping can be found in Appendix 3 of the full report.[1] A list of ANZCTR data fields and their definitions is available in Appendix 4 of the report.[1]

All analyses were conducted using the open-source software R and data outputs were cross-checked by a second, independent reviewer. The ANZCTR invested in developing semi-automated code to easily update all analyses and data outputs for future reports.

Data considerations

All data have been provided by the trial registrant, and the registrant is therefore responsible for their accuracy. The ANZCTR's review of submitted information helps to ensure content is complete and meaningful (as required by the WHO ICTRP) but this process cannot ensure that submitted information is accurate. Metrics at the ANZCTR indicate that around 65% to 77% of trials are updated at least once, with trial recruitment dates and sample size being the top 2 most frequently updated data fields.

The findings in the synopsis and report reflect data in the ANZCTR on 4 February 2021.

The data cover registered trials only and may not necessarily reflect overall trends in clinical trial activity. For example, any growth may be an artefact of increased trial registration, rather than increased trial activity.

Reference

1 Willson ML, Seidler AL, Aberoumand M, et al. Latest update of the clinical trials landscape in Australia 2006 – 2020. Sydney: Australian New Zealand Clinical Trials Registry, 2022. https://ses.library.usyd.edu.au/handle/2123/29703.2 (viewed May 2023).
